# Effects of Diabetes Mellitus on Corneal Immune Cell Activation and the Development of Keratopathy

**DOI:** 10.3390/cells13060532

**Published:** 2024-03-18

**Authors:** Pier Luigi Surico, Akitomo Narimatsu, Katayoon Forouzanfar, Rohan Bir Singh, Sara Shoushtari, Reza Dana, Tomas Blanco

**Affiliations:** Laboratory of Ocular Immunology, Transplantation and Regeneration, Schepens Eye Research Institute, Massachusetts Eye and Ear, Department of Ophthalmology, Harvard Medical School, Boston, MA 02114, USA; psurico@meei.harvard.edu (P.L.S.); anarimatsu@meei.harvard.edu (A.N.); kforouzanfar@meei.harvard.edu (K.F.); rohan_singh@meei.harvard.edu (R.B.S.); sara.shoushtari@meei.harvard.edu (S.S.); reza_dana@meei.harvard.edu (R.D.)

**Keywords:** diabetes mellitus, cornea, diabetic keratopathy, corneal nerves, myeloid cells

## Abstract

Diabetes mellitus (DM) is one of the most prevalent diseases globally, and its prevalence is rapidly increasing. Most patients with a long-term history of DM present with some degree of keratopathy (DK). Despite its high incidence, the underlying inflammatory mechanism of DK has not been elucidated yet. For further insights into the underlying immunopathologic processes, we utilized streptozotocin-induced mice to model type 1 DM (T1D) and B6.Cg-Lepob/J mice to model type 2 DM (T2D). We evaluated the animals for the development of clinical manifestations of DK. Four weeks post-induction, the total frequencies of corneal CD45^+^CD11b^+^Ly-6G^−^ myeloid cells, with enhanced gene and protein expression levels for the proinflammatory cytokines TNF-α and IL-1β, were higher in both T1D and T2D animals. Additionally, the frequencies of myeloid cells/mm^2^ in the sub-basal neural plexus (SBNP) were significantly higher in T1D and T2D compared to non-diabetic mice. DK clinical manifestations were observed four weeks post-induction, including significantly lower tear production, corneal sensitivity, and epitheliopathy. Nerve density in the SBNP and intraepithelial terminal endings per 40x field were lower in both models compared to the normal controls. The findings of this study indicate that DM alters the immune quiescent state of the cornea during disease onset, which may be associated with the progressive development of the clinical manifestations of DK.

## 1. Introduction

Diabetes mellitus (DM) is a chronic disorder affecting over half a billion individuals worldwide, and its prevalence is estimated to increase by 165% in the United States and more than 50% globally by 2050. A similar exponential increase is projected in the majority of developed and developing countries [1]. In the eye, DM affects the posterior and anterior ocular and adnexal tissues [2,3,4,5,6,7,8]. In the retina, DM induces neovascularization and microvascular damage, causing retinopathy [9,10,11,12], which is one of the leading causes of vision loss. DM is known to cause ocular surface changes, leading to a higher incidence of ocular surface disease, including dry eye, in diabetic patients [13,14,15,16]. Additionally, DM is also associated with other ophthalmic disorders, such as glaucoma [17,18] and cataracts [10,19].

Diabetic keratopathy (DK) is a chronic, sight-threatening condition affecting the cornea due to deviation from the normal wound-healing mechanisms due to corneal nerve damage and exhibits a spectrum of clinical manifestations, such as recurrent epithelial erosions, superficial punctate keratopathy, and decreased corneal sensitivity. These pathogenic changes are non-responsive to the available treatments and impact the quality of life [2,4,5,6,8,9,20,21,22]. Considering the rapidly increasing DM prevalence and high incidence of DK in patients with long-term disease [7,8,23,24], its prevalence will likely surpass all other ocular pathologies. Although DK patients have similar clinical presentations to neurotrophic keratopathy (NK) [25], the underlying pathological mechanisms in NK are exclusively neuropathic, whereas DK is primarily an inflammatory disorder and is caused by underlying immune cell-mediated mechanisms, which are not completely understood yet [26,27]. Therefore, it is pertinent to develop a better understanding of the immune cell-mediated pathological changes in the diabetic cornea and their association with the development of the clinical manifestations of DK. 

Corneal immune cells are bone marrow-derived CD45^+^CD11b^+^ myeloid immune cells that were first phenotyped and functionally characterized by our laboratory [28,29,30]. These cells share numerous functional properties with other bone marrow-derived myeloid cells. The corneal myeloid cell population has a predominantly immune-quiescent phenotype, i.e., low MHC-II, costimulatory molecule expression, and cytokine production [28,29]. However, these cells can acquire a highly mature and proinflammatory phenotype if stimulated, altering the corneal immune privilege [31,32].

Herein, we assessed the effect of DM on corneal myeloid cell infiltration and activation and the association with the development of the clinical manifestations of DK. In this work, we used both insulin-deficient STZ-induced type 1 diabetes (T1D) and insulin-resistant type 2 diabetes (T2D; Lep^ob/ob^) mouse models. We used well-established cellular and molecular techniques combined with intravital clinical imaging in vivo to assess the changes in the diabetic cornea and its relationship with the development of the clinical manifestations of DK. We observed increased myeloid cell infiltration with enhanced proinflammatory phenotypes in the corneas of both T1D and T2D mice during disease onset and enhanced clinical manifestations of DK.

## 2. Materials and Methods

### 2.1. Animals

Six-week-old male and female C57BL/6 mice (Charles River Laboratories, Wilmington, MA, USA) and 4-week-old male and female B6.Cg-Lep^ob^/J mice (Lep^ob/ob^, Jackson Laboratory, Bar Harbor, ME, USA) were used. All the animals were housed in a pathogen-free vivarium at the animal facility at Schepens Eye Research Institute of Massachusetts Eye and Ear (Protocol no. 2021N000247) and treated in strict compliance with the Association for Research in Vision and Ophthalmology Statement for the Use of Animals in Ophthalmic and Visual Research. All the experimental protocols were approved by the Institutional Animal Care and Use Committee. We used the corneas of six animals per group for all the experiments.

### 2.2. Diabetes Induction and Glycemic Control

**Figure 1:**
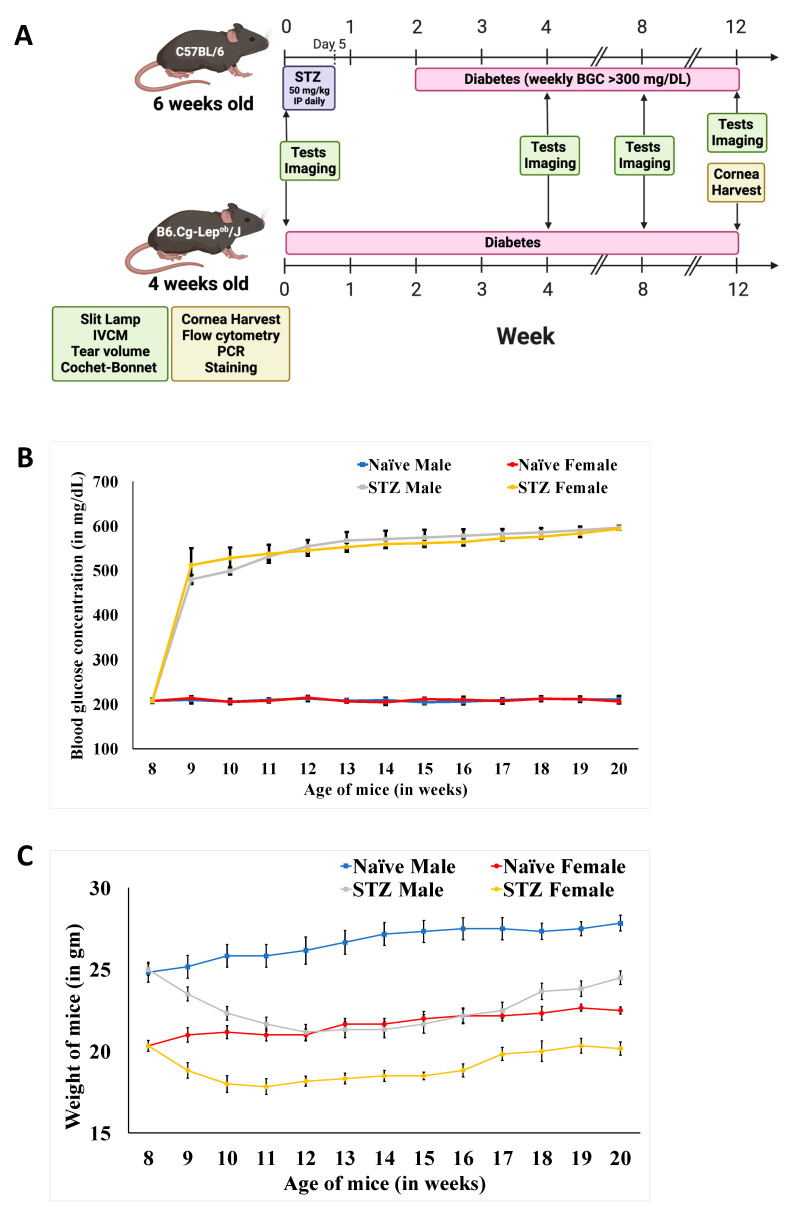
Diabetes-associated changes in mice. (**A**) Schematic diagram showing the induction of type 1 diabetes (T1D) with streptozotocin (STZ) injection in 8-week-old C57BL/6 mice that were followed up for 12 weeks. Once a week, (**B**) the blood glucose concentration (BGC) was measured, and (**C**) the animals were weighed. For type 2 diabetes (T2D), 4-week-old B6.Gg-Lepob/ob transgenic mice were used and followed up for 12 weeks. Normal, age-matched non-diabetic (ND) mice were used as the controls.

DM was induced in C57BL/6 mice (Figure 1A) via the daily intraperitoneal injection of 50 mg/kg streptozotocin (STZ) (Sigma-Aldrich GmbH, Steinheim, Germany) dissolved in 0.05 M sodium citrate buffer for five days, as performed previously [33]. At the same time, 4-week-old B6.Cg-Lep^ob^/J mice were purchased and incorporated into the vivarium. The mice were on a normal diet, and the blood glucose concentration (BGC) was measured once a week using a blood glucose monitor (Lifescan Inc., Milpitas, CA, USA) [34]. STZ-induced mice with a BGC of at least 11.1 mmol (300 mg/DL) for at least two consecutive weeks were considered diabetic. The animals were also weighed once a week. Lep^ob/ob^ mice are considered diabetic on arrival [35].

### 2.3. Corneal Single-Cell Suspensions 

The excised corneas were digested in RPMI-1640 media (Lonza Inc., Lexington, MA, USA) containing 2 mg/mL DNase I (Roche GmbH, Mannheim, Germany) and 0.5 mg/mL Collagenase D (Roche GmbH, Mannheim, Germany) for one hour at 37 °C and filtered through a 70-µm cell strainer. 

### 2.4. Flow Cytometry Analysis

The cell suspensions were incubated in phosphate-buffered saline with Zombie UV antibody (INDO-1/violet, 1:1000; BioLegend Inc., San Diego, CA, USA) for 20 min to differentiate the live cells from the non-viable cells, washed with 10% fetal bovine serum, and incubated with an Fc receptor-blocking antibody (R&D Systems, Minneapolis, MN, USA) for 20 min. The cells were stained with fluorescence-conjugated antibodies or isotype controls (Supplementary Table S1). The stained cells were analyzed using an LSRII flow cytometer (BD Biosciences Inc., San Jose, CA, USA), and the acquired data were analyzed using FlowJo software version 10.8.2 (FlowJo LLC, Ashland, OR, USA). 

### 2.5. Cell Sorting

CD45^+^CD11b^+^ cells were FACS-sorted from the single-cell suspensions derived from the corneoscleral tissue of diabetic and non-diabetic mice cells using a FACS sorter (BD FACSAria II; BD Biosciences Inc., San Jose, CA, USA). After sorting, the CD45^+^CD11b^+^ cells were stimulated with phorbol 12-myristate 13-acetate (PMA; 50 ng/mL; Sigma Aldrich Corp., St. Louis, MO, USA) and ionomycin (500 ng/mL; Sigma Aldrich Corp., St. Louis, MO, USA) for 6 h and the protein concentration of the cytokines (TNF-α, IL-1β; Invitrogen Inc., Carlsbad, CA, USA) in the supernatant was assessed via conducting an ELISA.

### 2.6. Polymerase Chain Reaction

The sorted cells were frozen in TRIzol (Invitrogen Inc., Carlsbad, CA, USA) at −80 °C. The total RNA was extracted using a commercially available kit, specifically the Rneasy Micro Kit (Qiagen Inc., Germantown, MD, USA), and reverse transcription was performed using the Super Script® III Kit (Invitrogen Inc., Carlsbad, CA, USA). To increase the sensitivity, three rounds of pre-amplification were performed, and a real-time polymerase chain reaction (qRT-PCR) was performed using Universal Taqman PCR Mastermix (Applied Biosystems Inc, Foster City, CA, USA) and FAM dye-labeled predesigned primers (Applied Biosystems Inc, Foster City, CA, USA) were used for the amplification of the genes expressing *TNF-α*, *IL-1β*, and *GAPDH*. The qRT-PCR was performed on an Eppendorf Mastercycler (Eppendorf North America, New York, NY, USA). The results were analyzed using the comparative threshold cycle method and normalized to *GAPDH* as an internal control.

### 2.7. Immunostaining and Confocal Microscopy

To examine the corneal nerve morphology and density, both the corneas of the mice (*n* = 6 mice per group) were carefully dissected and fixed in 4% paraformaldehyde solution for 40 min at room temperature. Before further processing, four radial relaxing incisions were performed in the corneas. Subsequently, the corneas were rinsed with PBS containing 0.1% bovine serum albumin (BSA) and blocked with a 1% BSA solution in PBS, along with 0.1% Triton X-100 for 2 h. The corneas were incubated with primary conjugated antibodies anti-β-tubulin-III NL557 (Tuj1, R&D System, Minneapolis, MN, USA) and anti-CD11b-FITC (BioLegend Inc., San Diego, CA, USA) (1:200) for 24 h at 4 °C. Subsequently, the corneas were rinsed with PBS and mounted in a medium containing DAPI (VectaShield; Vector Laboratories, Burlingame, CA, USA). The corneas were imaged using a confocal microscope (TCS-SP8, Leica Microsystems, Bannockburn, IL, USA). The nerve density and immune cells were quantified using ImageJ software version 1.54f with the NeuronJ plugin (National Institutes of Health, Bethesda, MD, USA). 

### 2.8. Tear Volume Assessment

Tear volume was determined via a phenol red thread test (PRTT) (FCI Ophthalmics, Tokyo, Japan) at baseline, 4, 8, and 12 weeks. For the tear volume assessment, we included both eyes of six animals per group. The tip thread was placed on the lateral cantus of the eye for 30 s, and the extent of color change was assessed as previously detailed [36].

### 2.9. Corneal Sensitivity Assessment

The corneal sensitivity was measured using a Cochet-Bonnet esthesiometer (Luneau Ophthalmologie, Paris, France), using nylon monofilaments with a maximal length of 60 mm and a diameter of 0.12 mm at baseline, 4, 8, and 12 weeks [36,37]. For the corneal nerve sensitivity assessment, we included both eyes of six animals per group. The central cornea of each eye was touched once using the full-length filaments, and the blinking response was interpreted as a positive result in the test. If a blinking response was not elicited, the length of the thread was progressively reduced by 5 mm each time until a blinking response was obtained. To ensure the reliability of the measurements, this process was repeated 5 times in each eye.

### 2.10. Corneal Epitheliopathy Assessment

After verifying the corneal transparency and the integrity of the anterior segment of the eye via a slit lamp examination, the corneal epitheliopathy was assessed using corneal fluorescein staining (CFS). Briefly, 1 μL of 2.5% fluorescein (Sigma-Aldrich GmbH, Steinheim, Germany) was administered to the lateral conjunctival sac of the mice, and the corneal examination was performed under cobalt blue light using a slit lamp. The assessment was performed in a blinded manner, and the epitheliopathy was graded using the National Eye Institute (NEI) system, grading punctate staining in each of the five corneal areas on a scale of 0 to 3—central, superior, inferior, nasal, and temporal—with a total score range of 0 to 15 per eye.

### 2.11. Optical Coherence Tomography

Optical coherence tomography (OCT) imaging was performed after slit lamp examination. As per our established protocol [38,39], the mice were anesthetized with ketamine/xylazine, and topical anesthesia using 0.5% proparacaine ophthalmic solution (1–2 drops) was applied to both eyes. For the evaluation of changes in the epithelial layer and central corneal thickness, we utilized an anterior segment optical coherence tomography (AS-OCT) system (Bioptigen Spectral Domain Ophthalmic Imaging System Envisu R2200) equipped with a 12-mm telecentric cornea lens. The changes in the central corneal thickness were quantified using the built-in software. 

### 2.12. In-Vivo Confocal Microscopy

In-vivo confocal microscopy (IVCM) was performed to assess the alterations in the central corneal anatomy, as performed in previous studies [40,41]. The mice were anesthetized with an intraperitoneal injection of ketamine/xylazine. Ophthalmic gel (GenTeal, Novartis, St. Louis, MO, USA) was used to create a coupling immersion interface with a refractive index (*n* = 1.339) similar to water (*n* = 1.333 at 20 °C) and for lubrication. The laser scanning Heidelberg Retina Tomograph III (HRT III) with the Rostock Corneal Module (RCM) (Heidelberg Engineering GmbH, Germany) with a diode laser with a 670 nm wavelength and a 60× objective immersion lens was used to perform the imaging of both the eyes. The images were obtained in an area of 400 × 400 μm, with a transverse optical resolution of approximately 1 mm/pixel of all the different layers of the cornea. The epithelium and sub-basal nerves were subjectively evaluated, while both the subepithelial dendritic cell and endothelial cell density (cells/mm^2^) were quantified in each frame using built-in software [38,39].

### 2.13. Statistical Analyses

Prism 9.5.1 (GraphPad Software, San Diego, CA, USA) was used for the statistical analysis, and ImageJ (National Institutes of Health, Bethesda, MD, USA) was used for cell quantification and nerve density analysis. A pre-study power analysis was performed, and six mice per group were included to achieve a statistical significance of 0.80. A significance of 0.80 was determined using G*Power 3.1 analysis using the following parameters: F tests, ANOVA: fixed effects, special, main effects, and interactions, an effective size f of 2 (based on partial η^2^ of 0.8), a numerator df of 2 (k − 1), and the number of groups set to 3 (k = 3). The two-tailed student’s t-test with Sidak’s correction was used for the quantitative data analysis between the two groups. An analysis of variance (ANOVA) with a Bonferroni correction was performed to determine the differences between the means of three or more independent (unrelated) groups. The results are presented as the mean ± standard error of the mean (SEM). The *p*-values of ≤0.05 were considered statistically significant.

## 3. Results

### 3.1. Weight and Blood Glucose Concentration Changes on Diabetes Induction in Mice

Following STZ-induction, we assessed the blood glucose concentration (BGC) and body weight to confirm T1D onset. Although a sustained BGC over 300 mg/dL is categorized as hyperglycemia, we observed the BGC increased to ~400 mg/dL during week 1 (Figure 1B). At 5 weeks post-induction, all the mice had a BGC higher than the maximum (600 mg/dL) detected using the glucometer, and no difference was observed between the male and female mice (*n* = 12/group). The STZ treatment resulted in a significant weight loss in the first four weeks post-induction compared to non-diabetic (ND) mice (Figure 1C). Thereafter, T1D mice had a steady weight, which moderately increased with aging. The relatively high drop in the weight of diabetic males compared to females was due to higher resistance to the diabetogenic effects of STZ in the latter [42]. The significant increase in the BGC following STZ induction and the decreased weight demonstrate the development of T1D in STZ-treated mice. For modeling T2D, we used Lep^ob/ob^ mice that underwent transient hyperglycemia at 8 weeks, then the BGC progressively reduced until the mice were 16 weeks old and presented normoglycemia. These changes are well-documented by the vendor providing the mice [43]. However, the body weight was significantly increased (up to 60 g) in 16-week-old mice [43]. Considering the well-being of the obese animals, we established 16 weeks as the endpoint for the experiments with Lep^ob/ob^ mice.

### 3.2. Impact of Diabetes on the Corneal Anatomy and Cellular Morphology 

**Figure 2:**
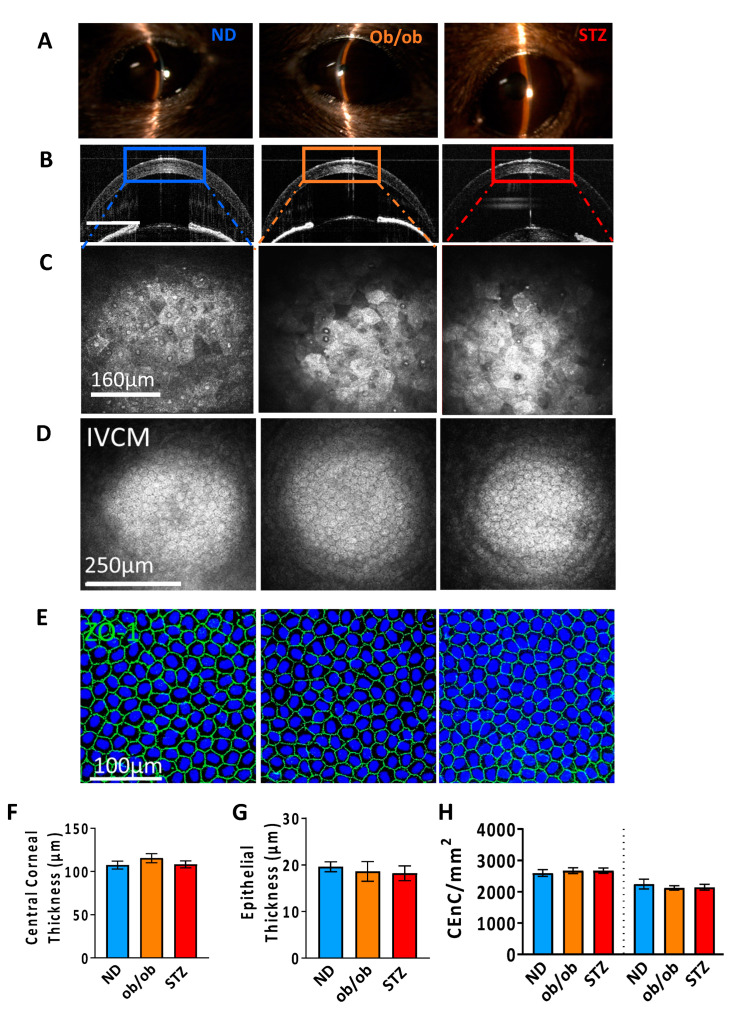
Assessment of gross and cellular changes in the diabetic corneas. (**A**) Corneal transparency was evaluated via slit lamp biomicroscopy. (**B**) Changes in the corneal thickness were intravitally imaged using anterior segment optical coherence tomography (AS-OCT). The HRT-3-IVCM system was used to intravitally evaluate the different layers of the cornea, including the (**C**) epithelium and (**D**) endothelium. (**E**) ZO-1 staining was performed in 4% PFA fixed corneal explants, then imaged with a confocal microscope, and ZO-1 cells/mm^2^ were counted in the center of the cornea. The bar graphs represent the (**F**) central corneal total thickness (CCT), (**G**) epithelial thickness at 12 weeks, and (**H**) corneal endothelial cell (CEnC) density at baseline and week 12. ND: normal non-diabetic mice; ob/ob: type 2 diabetic B6.Cg-Lepob/J transgenic mice; STZ: STZ-induced type 1 diabetic mice; *n* = 6 mice/group.

The diabetic mice were evaluated for anatomical and morphological changes in the cornea. We did not observe any changes in the corneal transparency or neovascularization upon slit lamp examination in either the diabetic or non-diabetic mice (Figure 2A). Additionally, no evidence of inflammatory cell infiltration was observed in the anterior chamber upon examination, which was further confirmed via AS-OCT (Figure 2B). However, the corneal epithelium appeared hyperreflective in diabetic mice, indicating cell death in the apical stratum (as observed via IVCM) compared to non-diabetic mice (Figure 2C). We did not detect significant differences in the CEnC density in age-matched diabetic and non-diabetic mice (Figure 2D,E,H). The total central corneal and epithelial thicknesses were comparable between both of the groups (Figure 2F,G). These observations indicate that diabetes does not impact the corneal anatomy; however, the disease induces cell death in the corneal epithelium.

### 3.3. A Diabetic State Induces Corneal Myeloid Cells to Acquire a Proinflammatory Phenotype in Myeloid Cells

**Figure 3:**
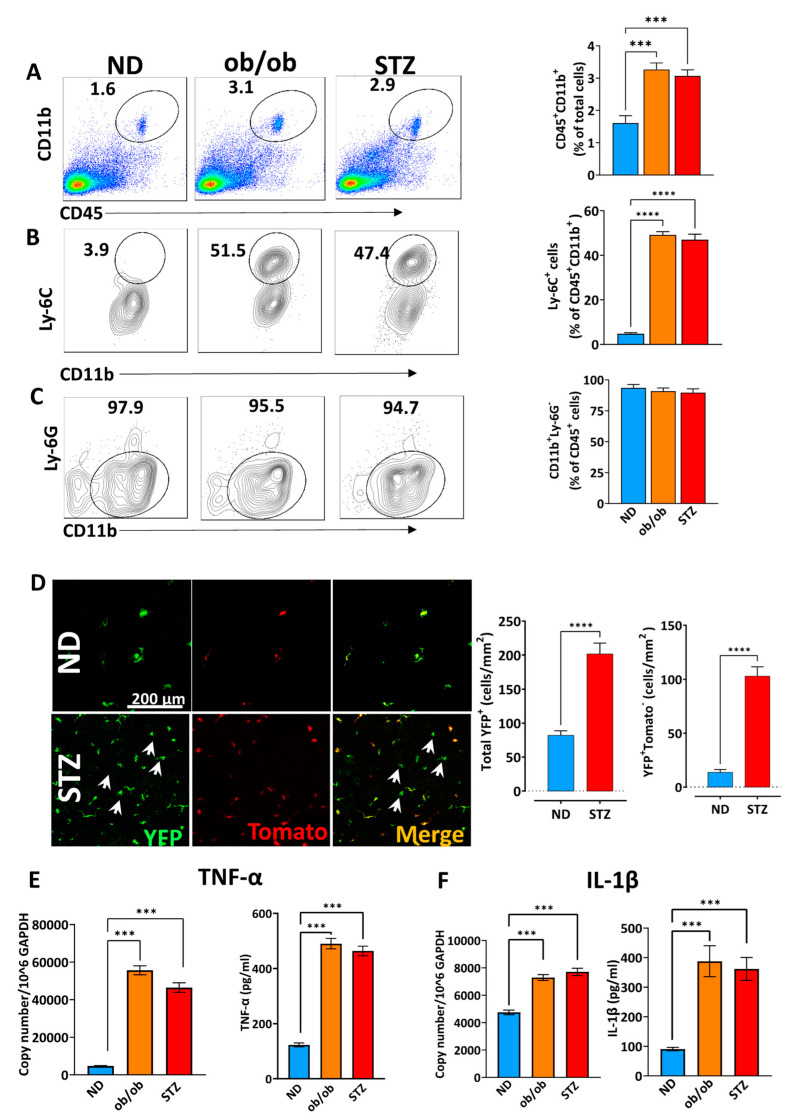
Changes in the immune cell frequencies of diabetic mice. At 12 weeks, the animals were sacrificed, and the corneas were harvested, which were digested with collagenase and DNase. The myeloid cells in the harvested tissues were analyzed using flowcytometry. (**A**) CD45^+^CD11b^+^, (**B**) Ly-6C^+^CD11b^+^, and (**C**) lack of neutrophilic infiltration was observed. (**D**) STZ-induced Cx3Cr1^YFP^Cre^tomato^ T1D mice were used to confirm the infiltration of myeloid cells (arrows) in the cornea. Before inducing DM, the animals were injected with tamoxifen to induce the expression of the red protein tdTomato in all the Cx3Cr1^YFP+^-expressing myeloid cells in the cornea (double positive, orange). Afterward, only myeloid cells (YFP^+^tdTomato^−^) that infiltrate the cornea after DM induction are shown in green. The corneas of 10 mice (week 4) in each group were pooled, and the CD45^+^CD11b^+^ cells were FACS-sorted and assessed via qRT-PCR and ELISA to evaluate the expression of the proinflammatory cytokines (**E**) TNF-α and (**F**) IL-1β. ND: normal non-diabetic mice; ob/ob: type 2 diabetic B6.Cg-Lepob/J transgenic mice; STZ: STZ-induced type 1 diabetic mice; *n* = 6 mice/group. *** *p* < 0.001, **** *p* < 0.0001.

The normal cornea has a heterogeneous population of myeloid immune cells that are typically in a quiescent state [31]; however, the changes in the ocular surface milieu activate these immune cells [31,32]. As highlighted in the previous sections, there were no changes in the corneal anatomy over three months post-DM induction. It is well established that DM significantly impacts epithelial integrity [27], which may be associated with alterations in the immune population. Therefore, we investigated changes in the myeloid immune population post-DM induction in mice. The frequencies of myeloid cells profiled as CD45^+^CD11b^+^ were significantly increased in the corneas derived from T1D and T2D mice compared to the ND controls. (Figure 3A) For the flow cytometry analysis, we observed that these cells were predominantly Ly-6C^+^ (Figure 3B) and had no neutrophilic (Ly6G^+^) signature (Figure 3C). Moreover, we corroborated these findings with STZ-induced Cx3Cr1^YFP^Cre^tomato^ T1D mice. We observed increased myeloid cell infiltration throughout the corneal tissue. However, it was more evident in the center in the diabetic mice compared to the non-diabetic controls (Figure 3D).

Since corneal myeloid cells CD45^+^CD11b^+^ DC and other subsets (such as macrophages and Langerhans cells) are known to initiate the innate and adaptive immune responses [29,30,44], we specifically sorted CD45^+^CD11b^+^Ly-6G^−^ cells to assess the expression of proinflammatory cytokines [29,30,44]. Upon performing qRT-PCR and an ELISA, we observed significantly higher expression of prototypical inflammatory cytokines TNF-α (Figure 3E) and IL-1β (Figure 3F) in myeloid cells isolated from the diabetic samples compared to non-diabetic corneas. Collectively, these results confirm that DM not only promotes myeloid infiltration but also induces the activation of immune cells into a proinflammatory phenotype. 

### 3.4. Diabetic State-Induced Corneal Nerve Damage Is Associated with Long-Term Diabetes

**Figure 4:**
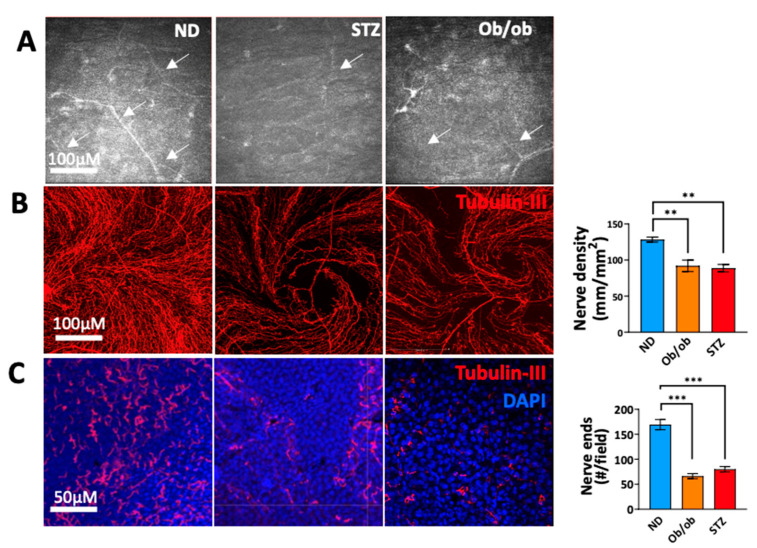
Diabetes induces corneal nerve damage. (**A**) Corneal sub-basal nerves (arrows) were imaged in vivo using an HRT-3-IVCM system. At 12 weeks, the corneas were harvested, fixed, and stained with anti-tubulin-III (a pan-neural marker). The nerve density in the sub-basal nerve plexus (SBNP) (**B**) and the number of intraepithelial terminal endings (**C**) in each group are represented. Tubulin-III (red) DAPI (blue). ND: normal non-diabetic mice; ob/ob: type 2 diabetic B6.Cg-Lepob/J transgenic mice; STZ: STZ-induced type 1 diabetic mice; *n* = 6 mice/group. ** *p* < 0.01, *** *p* < 0.001.

DM has been associated with a decrease in corneal nerve density. Therefore, we performed follow-ups on the mice using an HRT-3-IVCM imaging system. We observed a moderate reduction in the nerve density in the sub-basal nerve plexus (SBNP) at 6–8 weeks. However, this change was significant at 12 weeks in diabetic mice compared to the non-diabetic controls. (Figure 4A), This was further confirmed via the anti-tubulin-III (pan-neural marker) staining of the corneal explants, which showed significantly reduced nerve density (Figure 4B) and intraepithelial terminal endings (Figure 4C) in the SBNP of the corneas derived from diabetic mice compared the non-diabetic controls. Interestingly, the densities of the stromal nerves were comparable in diabetic and non-diabetic mice. These results indicate an association between long-term nerve damage and a higher expression of proinflammatory cytokines by myeloid cells during disease onset.

### 3.5. A Diabetic State Leads to Increased Frequencies of Myeloid Cells in the Proximity of Nerves in the Sub-Basal Plexus

**Figure 5:**
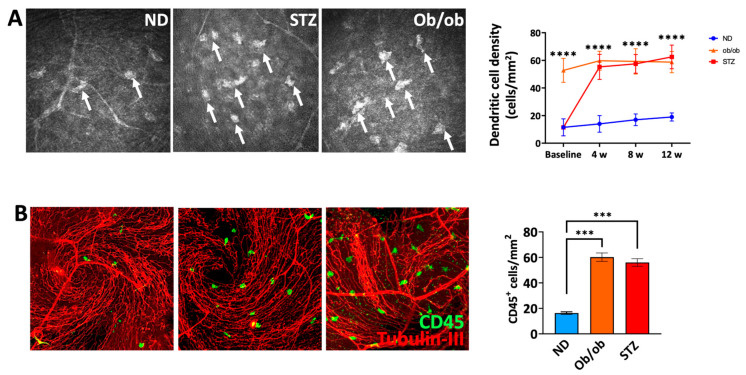
Distribution of myeloid cells within the sub-basal nerve plexus in diabetic mice. (**A**) Corneal sub-basal immune hyperreflective immune cells (arrows) were imaged in vivo using an HRT-3-IVCM system as above. (**B**) At 12 weeks, the corneas were harvested, fixed, and stained with anti-tubulin-III (a pan-neural marker) (red) and anti-CD11b (green) antibodies and imaged with a confocal microscope. The density of CD11b^+^ cells in the sub-basal-nerve plexus is shown. ND: normal non-diabetic mice; ob/ob: type 2 diabetic B6.Cg-Lepob/J transgenic mice; STZ: STZ-induced type 1 diabetic mice; *n* = 6 mice/group. *** *p* < 0.001, **** *p* < 0.0001.

In the corneal tissue, myeloid cells are in proximity to nerves in the SBNP and crosstalk is essential for the maintenance of the primary functions of the tissue, and any alterations can potentially jeopardize it [45,46,47]. Thus, we investigated the alterations in the myeloid cell density in diabetic mice in proximity to the nerves in the SBNP. Intravital imaging showed a significantly higher density of subepithelial immune cells in both T1D and T2D mice compared to the non-diabetic controls during disease onset (Figure 5A), which was further confirmed ex vivo via confocal microscopy (Figure 5B). These suggest that the amplified recruitment of proinflammatory immune cells observed in SBNP in the diabetic state may play a role in neuroinflammation and damage. 

### 3.6. Diabetic Mice with Long-Term DM Develop Clinical Manifestations of Keratopathy

**Figure 6:**
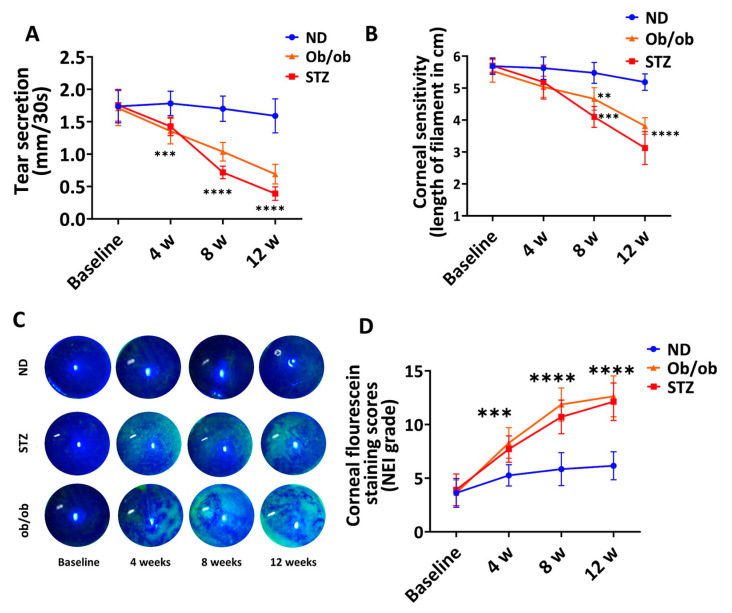
Evaluation of the clinical manifestations of keratopathy in diabetic mice. The changes in the ocular surface parameters were assessed. (**A**) Tear production was measured via the phenol red thread test for 30 s. (**B**) Corneal sensitivity was assessed by measuring the filament length using a Cochet-Bonnet esthesiometer. (**C**) Representative slit-lamp images and (**D**) grading highlighting different degrees of corneal epitheliopathy as assessed via corneal fluorescein staining (CFS). ND: normal non-diabetic mice; ob/ob: type 2 diabetic B6.Cg-Lepob/J transgenic mice; STZ: STZ-induced type 1 diabetic mice; *n* = 6 mice/group. ** *p* < 0.01. *** *p* < 0.001. **** *p* < 0.0001.

Lastly, we evaluated the clinical manifestations of keratopathy in diabetic mice. We observed a progressive reduction in the tear volume (mm/30 s; measured via the phenol red thread test) in both the STZ-induced and Lep^ob/ob^ mice. The tear volume was significantly lower in diabetic mice 4 weeks post-induction compared to the non-diabetic controls (Figure 6A). The corneal sensitivity (evaluated with a Cochet-Bonnet esthesiometer) progressively decreased during the study period and was significantly lower in both T1D and T2D mice at 8 weeks compared to the non-diabetic mice (Figure 6B). The corneal epitheliopathy, assessed via corneal fluorescein staining (CFS) and graded as per the standardized NEI (0–15) grading system, was observed to be significantly higher at 4, 8, and 12 weeks in the diabetic mice compared to the non-diabetic mice (Figure 6C,D). These results highlight that the development of the worsening clinical signs and manifestations of DK are associated with long-term diabetes associated with keratopathy in both T1D and T2D mice during disease onset and further aggravates DM progression. 

## 4. Discussion

The cornea is a unique tissue due to its constant exposure to pathogens, foreign antigens, and allergens, often without eliciting immune responses; these properties are known as corneal immune privilege. This immune privilege is attributed to an array of factors, including the immature state of the myeloid cells [28,29]. The diabetic state leads to the breakdown of this quiescent immune state through the induction of an immunostimulatory phenotype in corneal myeloid cells [47]. These cells reside in close proximity to the sub-basal nerves [47], and neuronal–immune crosstalk is essential for the tissue’s primary functions [45,47,48,49]. Previous studies have highlighted the role of myeloid cells in delayed wound healing [50].

The immune-mediated damage to corneal nerves leads to a reduction in mechanical sensitivity, which is a common corneal presentation in DM patients [7,8,23,24]. The proinflammatory milieu in diabetic corneas is primarily attributed to the accumulation of advanced glycation end-products (AGEs) [27], highly expressed proinflammatory molecules observed in hyperglycemic states. AGEs are known to activate nuclear factor kappa beta (NF-kB) in immune cells [51,52,53], resulting in elevated expression levels of proinflammatory cytokines and chemokines [54,55]. Although previous studies have associated corneal nerve damage in diabetic patients with AGE accumulation and oxidative stress [21], the precise underlying mechanisms are not understood completely. 

Herein, we present evidence that associates nerve damage with the increased frequency of proinflammatory myeloid cells in diabetic corneas. For the first time, we report that the nerve damage observed in diabetes is primarily caused by increased frequencies of corneal myeloid cells that acquire an immunostimulatory phenotype expressing high levels of proinflammatory TNF-α and IL-1β, which is in line with our previous work using a corneal transplantation model with diabetic donors [33]. These myeloid cells accumulate near the proximity of the nerves in the SBNP during the onset of the disease, similar to those shown in other models of corneal inflammation [45], and explain the nerve damage observed in this work. We observed increased myeloid cell infiltration across the corneal tissue, with a particularly pronounced presence in the central region among diabetic mice in contrast to non-diabetic controls. This observation aligns with the increased density of myeloid cells in the central cornea observed in individuals with type 2 diabetes mellitus (T2DM) and chronic kidney disease (CKD) who are experiencing central corneal nerve loss [56].

In addition to epitheliopathy, all diabetic mice showed decreased tear production and sensitivity. The high frequencies of myeloid cells in diabetic mice and the proinflammatory phenotypic changes in these cells were observed during the onset of the disease (1–4 weeks after induction), whereas nerve damage and clinical manifestations were observed in the later stages. We can speculate the critical role of myeloid cells in inducing clinical changes one month post-T1D induction (with STZ) and in 8-week-old T2D mice. Although there is no clear onset period in our T2D models, the highest frequencies of myeloid cells with an enhanced proinflammatory profile were observed at 8 weeks of life. Although both models exhibited distinct timelines for cellular and clinical changes associated with diabetes, both mirrored the observed results. 

In the experiments outlined in this report, we used both STZ-induced T1D [33,34,57,58] and Lep^ob/ob^ mice T2D models [59,60,61,62,63]. STZ is a β-cell-specific toxin that induces irreversible damage to pancreatic islets, causing insulin deficiency and chronic hyperglycemia, whereas Lep^ob/ob^ mice contain a mutation in the leptin gene and exhibit obesity, glucose intolerance with transient hyperglycemia, and hyperinsulinemia with insulin resistance. Although there is high variability in the pathogenesis of T1D and T2D, both forms of the disease have common fundamental mechanisms for leukocyte maturation [33]. That evidence suggests that the corneal complications are similar in either type of disease [27], and our data show no differences in these models as well. The prevalence of T2D (90%) is significantly higher than T1D (5.8%), which is reflected in the total number of patients with a history of T2D showing clinical manifestations of DK [64]. However, since these underlying immunological mechanisms are similar, either model can be used for further investigations indistinctively.

Transient hyperglycemia is a potential limitation in the use of Lep^ob/ob^ mice for mimicking T2D. In these mice, the BGC peaks at 8 weeks post-birth and then progressively declines to normoglycemic levels by week 16. However, our results indicate that once myeloid cells are reprogrammed in these animals, the cells maintain the proinflammatory phenotype even after a decrease in the BGC. This is consistent with our previous work in which we showed the increased immunostimulatory phenotype in corneal myeloid cells in 16-week-old Lep^ob/ob^ mice [33]. Similarly, in STZ-induced DM mice, immune cells quickly acquire an immunostimulatory profile that remains persistent unless the animals are treated with insulin [33]. Obesity is a second major limitation in the use of Lep^ob/ob^ mice. The 16-week-old mice weighed approximately 60 g, and it is not recommended that it be continued for experiments with longer follow-up durations. However, considering that during the mice’s adulthood, 12.26 mouse days is equivalent to one human year [65], a 12-week-old mouse has an equivalence of seven human years. Considering that the onset of diabetes has been estimated to occur in four to seven years in humans [66,67], we can conclude that 12 weeks is long-term diabetes in mice and, therefore, deduced to humans.

Evidence in the literature suggests that female mice are more resistant to low doses of STZ treatment, and therefore, the majority of the published work in this area is in males [34,68,69,70,71,72]. In this study, we used both males and females with an increased dose of STZ (50 g/kg), showing similar BGCs and weight loss as shown more recently by other authors with similar dosing [34,73,74,75]. Moreover, we did not find significant differences in leukocyte infiltration, nerve damage, or clinical manifestations between both the sexes, and therefore, all mice were pooled together irrespective of their sex. Some studies have shown that females with DM are more prone to diabetic peripheral neuropathies (DPNs) [76]; however, no differences were observed in the DPN onset age and diabetes duration (before DPN onset) in both sexes. Moreover, there is no evidence suggesting variation in DK prevalence between males and females [20].

## 5. Conclusions

In conclusion, we observed a higher density of myeloid cells in the corneas of T1D and T2D mice, as well as an increase in their proinflammatory signatures. In T1D and T2D mice, myeloid cell infiltration in the SBNP potentially leads to neuronal damage and reduced density. Both T1D and T2D mice developed similar clinical manifestations of DK, demonstrated by their impaired sensitivity, low tear production, and epithelial erosions. With the rapidly increasing prevalence of diabetes, these models represent a reliable method for assessing the cellular and molecular immune mechanisms driving the development of DK as well as future investigations of other DM-associated ocular surface pathologies.

## Data Availability

The data reported in this study are available from the corresponding author (T.B.) upon reasonable request.

## Supplementary Materials

The following supporting information can be downloaded at: https://www.mdpi.com/article/10.3390/cells13060532/s1, Table S1: Flow cytometry antibodies used in the experiments.

## References

[1] Saeedi P., Petersohn I., Salpea P., Malanda B., Karuranga S., Unwin N., Colagiuri S., Guariguata L., Motala A.A., Ogurtsova K. (2019). Global and Regional Diabetes Prevalence Estimates for 2019 and Projections for 2030 and 2045: Results from the International Diabetes Federation Diabetes Atlas, 9th Edition. Diabetes Res. Clin. Pract..

[2] Sayin N., Kara N., Pekel G. (2015). Ocular Complications of Diabetes Mellitus. World J. Diabetes.

[3] DeMill D.L., Hussain M., Pop-Busui R., Shtein R.M. (2016). Ocular Surface Disease in Patients with Diabetic Peripheral Neuropathy. Br. J. Ophthalmol..

[4] Vieira-Potter V.J., Karamichos D., Lee D.J. (2016). Ocular Complications of Diabetes and Therapeutic Approaches. Biomed. Res. Int..

[5] Lutty G.A. (2013). Effects of Diabetes on the Eye. Investig. Ophthalmol. Vis. Sci..

[6] Semeraro F., Cancarini A., Dell’Omo R., Rezzola S., Romano M.R., Costagliola C. (2015). Diabetic Retinopathy: Vascular and Inflammatory Disease. J. Diabetes Res..

[7] Yu F.S., Yin J., Lee P.S., Hwang F.S., McDermott M. (2015). Sensory Nerve Regeneration after Epithelium Wounding in Normal and Diabetic Corneas. Expert. Rev. Ophthalmol..

[8] Kaji Y. (2005). Prevention of Diabetic Keratopathy. Br. J. Ophthalmol..

[9] Negi A., Stephen M., Vernon A., Frcophth D.M. (2003). An Overview of the Eye in Diabetes. J. R. Soc. Med..

[10] Klein B.E.K., Klein R., Moss S.E. (1995). Incidence of Cataract Surgery in the Wisconsin Epidemiologic Study of Diabetic Retinopathy. Am. J. Ophthalmol..

[11] Messmer E.M., Schmid-Tannwald C., Zapp D., Kampik A. (2010). In Vivo Confocal Microscopy of Corneal Small Fiber Damage in Diabetes Mellitus. Graefe’s Arch. Clin. Exp. Ophthalmol..

[12] Nitoda E., Kallinikos P., Pallikaris A., Moschandrea J., Amoiridis G., Ganotakis E.S., Tsilimbaris M. (2012). Correlation of Diabetic Retinopathy and Corneal Neuropathy Using Confocal Microscopy. Curr. Eye Res..

[13] Naik K., Magdum R., Ahuja A., Kaul S., Johnson S., Mishra A., Patil M., Dhore N., Alapati A. (2022). Ocular Surface Diseases in Patients with Diabetes. Cureus.

[14] Zhang Z., Zou X., Xue W., Zhang P., Wang S., Zou H. (2021). Ocular Surface Microbiota in Diabetic Patients with Dry Eye Disease. Investig. Ophthalmol. Vis. Sci..

[15] Bu Y., Shih K.C., Tong L. (2022). The Ocular Surface and Diabetes, the Other 21st Century Epidemic. Exp. Eye Res..

[16] Di Zazzo A., Coassin M., Micera A., Mori T., De Piano M., Scartozzi L., Sgrulletta R., Bonini S. (2021). Ocular Surface Diabetic Disease: A Neurogenic Condition?. Ocul. Surf..

[17] Chopra V., Varma R., Francis B.A., Wu J., Torres M., Azen S.P. (2008). Type 2 Diabetes Mellitus and the Risk of Open-Angle Glaucoma: The Los Angeles Latino Eye Study. Ophthalmology.

[18] Saw S.M., Wong T.Y., Ting S., Foong A.W.P., Foster P.J. (2007). The Relationship Between Anterior Chamber Depth and the Presence of Diabetes in the Tanjong Pagar Survey. Am. J. Ophthalmol..

[19] Huang S.P., Palla S., Ruzycki P., Varma R.A., Harter T., Reddy G.B., Petrash J.M. (2010). Aldo-Keto Reductases in the Eye. J. Ophthalmol..

[20] Priyadarsini S., Whelchel A., Nicholas S., Sharif R., Riaz K., Karamichos D. (2020). Diabetic Keratopathy: Insights and Challenges. Surv. Ophthalmol..

[21] Shih K.C., Lam K.L., Tong L. (2017). A Systematic Review on the Impact of Diabetes Mellitus on the Ocular Surface. Nutr. Diabetes.

[22] Han S.B., Yang H.K., Hyon J.Y. (2019). Influence of Diabetes Mellitus on Anterior Segment of the Eye. Clin. Interv. Aging.

[23] Sellers E.A.C., Clark I., Tavakoli M., Dean H.J., McGavock J., Malik R.A. (2013). The Acceptability and Feasibility of Corneal Confocal Microscopy to Detect Early Diabetic Neuropathy in Children: A Pilot Study. Diabet. Med..

[24] Barsegian A., Lee J., Salifu M.O., Mcfarlane S.I. (2018). Corneal Neuropathy: An Underrated Manifestation of Diabetes Mellitus. J. Clin. Endocrinol. Diabetes.

[25] NaPier E., Camacho M., McDevitt T.F., Sweeney A.R. (2022). Neurotrophic Keratopathy: Current Challenges and Future Prospects. Ann. Med..

[26] Zhou Q., Yang L., Wang Q., Li Y., Wei C., Xie L. (2022). Mechanistic Investigations of Diabetic Ocular Surface Diseases. Front. Endocrinol..

[27] Ljubimov A.V. (2017). Diabetic Complications in the Cornea. Vis. Res..

[28] Hamrah P., Liu Y., Zhang Q., Dana M.R. (2003). The Corneal Stroma Is Endowed with a Significant Number of Resident Dendritic Cells. Investig. Ophthalmol. Vis. Sci..

[29] Hamrah P., Liu Y., Zhang Q., Dana M.R. (2003). Alterations in Corneal Stromal Dendritic Cell Phenotype and Distribution in Inflammation. Arch. Ophthalmol..

[30] Hamrah P., Zhang Q., Liu Y., Dana M.R. (2002). Novel Characterization of MHC Class II-Negative Population of Resident Corneal Langerhans Cell-Type Dendritic Cells. Investig. Ophthalmol. Vis. Sci..

[31] Amouzegar A., Chauhan S.K., Dana R. (2016). Alloimmunity and Tolerance in Corneal Transplantation. J. Immunol..

[32] Huq S., Liu Y., Benichou G., Dana M.R. (2004). Relevance of the Direct Pathway of Sensitization in Corneal Transplantation Is Dictated by the Graft Bed Microenvironment. J. Immunol..

[33] Blanco T., Musayeva A., Singh R.B., Nakagawa H., Lee S., Alemi H., Gonzalez-Nolasco B., Ortiz G., Wang S., Kahale F. (2023). The Impact of Donor Diabetes on Corneal Transplant Immunity. Am. J. Transplant..

[34] Leppin K., Behrendt A.K., Reichard M., Stachs O., Guthoff R.F., Baltrusch S., Eule J.C., Vollmar B. (2014). Diabetes Mellitus Leads to Accumulation of Dendritic Cells and Nerve Fiber Damage of the Subbasal Nerve Plexus in the Cornea. Investig. Ophthalmol. Vis. Sci..

[35] 000632-B6 Ob Strain Details. https://www.jax.org/strain/000632.

[36] De Silva M.E.H., Hill L.J., Downie L.E., Chinnery H.R. (2019). The Effects of Aging on Corneal and Ocular Surface Homeostasis in Mice. Investig. Ophthalmol. Vis. Sci..

[37] Stepp M.A., Pal-Ghosh S., Tadvalkar G., Williams A., Pflugfelder S.C., de Paiva C.S. (2018). Reduced Intraepithelial Corneal Nerve Density and Sensitivity Accompany Desiccating Stress and Aging in C57BL/6 Mice. Exp. Eye Res..

[38] Alemi H., Dehghani S., Forouzanfar K., Surico P.L., Narimatsu A., Musayeva A., Sharifi S., Wang S., Dohlman T.H., Yin J. (2023). Insights into Mustard Gas Keratopathy- Characterizing Corneal Layer-Specific Changes in Mice Exposed to Nitrogen Mustard. Exp. Eye Res..

[39] Alemi H., Wang S., Blanco T., Kahale F., Singh R.B., Ortiz G., Musayeva A., Yuksel E., Pang K., Deshpande N. (2023). The Neuropeptide α-Melanocyte–Stimulating Hormone Prevents Persistent Corneal Edema Following Injury. Am. J. Pathol..

[40] Blanco-Mezquita J.T., Hutcheon A.E.K., Zieske J.D. (2013). Role of Thrombospondin-1 in Repair of Penetrating Corneal Wounds. Investig. Ophthalmol. Vis. Sci..

[41] Tatematsu Y., Khan Q., Blanco T., Bair J.A., Hodges R.R., Masli S., Dartt D.A. (2018). Thrombospondin-1 Is Necessary for the Development and Repair of Corneal Nerves. Int. J. Mol. Sci..

[42] Saadane Id A., Lessieur E.M., Du Y., Liu H., Kern T.S. (2020). Successful Induction of Diabetes in Mice Demonstrates No Gender Difference in Development of Early Diabetic Retinopathy. PLoS ONE.

[43] https://Jackson.Jax.Org/Rs/444-BUH-304/Images/632%20Physiological%20Data%20Summary.Pdf.

[44] Liu Y., Hamrah P., Zhang Q., Taylor A.W., Reza Dana M. (2002). Draining Lymph Nodes of Corneal Transplant Hosts Exhibit Evidence for Donor Major Histocompatibility Complex (MHC) Class II-Positive Dendritic Cells Derived from MHC Class II-Negative Grafts. J. Exp. Med..

[45] Wu M., Hill L.J., Downie L.E., Chinnery H.R. (2022). Neuroimmune Crosstalk in the Cornea: The Role of Immune Cells in Corneal Nerve Maintenance during Homeostasis and Inflammation. Prog. Retin. Eye Res..

[46] Jiao H., Lim A.S., Fazio Coles T.E., McQuade R.M., Furness J.B., Chinnery H.R. (2020). The Effect of High-Fat Diet-Induced Metabolic Disturbance on Corneal Neuroimmune Features. Exp. Eye Res..

[47] Blanco T., Saban D.R. (2015). The Cornea Has “the Nerve” to Encourage Immune Rejection. Am. J. Transplant..

[48] Krishna Kolluru G., Bir S.C., Kevil C.G., Calvert J.W. (2012). Endothelial Dysfunction and Diabetes: Effects on Angiogenesis, Vascular Remodeling, and Wound Healing. Int. J. Vasc. Med..

[49] Frutos-rincón L., Gómez-sánchez J.A., Íñigo-Portugués A., Carmen Acosta M., Gallar J. (2022). An Experimental Model of Neuro–Immune Interactions in the Eye: Corneal Sensory Nerves and Resident Dendritic Cells. Int. J. Mol. Sci..

[50] Joshi N., Pohlmeier L., Ben-Yehuda Greenwald M., Haertel E., Hiebert P., Kopf M., Werner S. (2020). Comprehensive Characterization of Myeloid Cells during Wound Healing in Healthy and Healing-Impaired Diabetic Mice. Eur. J. Immunol..

[51] Kim J., Kim C.S., Sohn E., Jeong I.H., Kim H., Kim J.S. (2011). Involvement of Advanced Glycation End Products, Oxidative Stress and Nuclear Factor-KappaB in the Development of Diabetic Keratopathy. Graefe’s Arch. Clin. Exp. Ophthalmol..

[52] Alves M., Cunha D.A., Calegari V.C., Saad M.J.A., Boschero A.C., Velloso L.A., Rocha E.M. (2005). Nuclear Factor-KappaB and Advanced Glycation End-Products Expression in Lacrimal Glands of Aging Rats. J. Endocrinol..

[53] Lan W., Petznick A., Heryati S., Rifada M., Tong L. (2012). Nuclear Factor-ΚB: Central Regulator in Ocular Surface Inflammation and Diseases. Ocul. Surf..

[54] Yan C., Gao N., Sun H., Yin J., Lee P., Zhou L., Fan X., Yu F.-S. (2016). Targeting Imbalance between IL-1b and IL-1 Receptor Antagonist Ameliorates Delayed Epithelium Wound Healing in Diabetic Mouse Corneas. Am. J. Pathol..

[55] Di Zazzo A., Coassin M., Surico P.L., Bonini S. (2022). Age-Related Ocular Surface Failure: A Narrative Review. Exp. Eye Res..

[56] Asiedu K., Markoulli M., Tummanapalli S.S., Chiang J.C.B., Alotaibi S., Wang L.L., Dhanapalaratnam R., Kwai N., Poynten A., Krishnan A.V. (2023). Impact of Chronic Kidney Disease on Corneal Neuroimmune Features in Type 2 Diabetes. J. Clin. Med..

[57] Zhao L., Li Y., Lv Q., Wang M., Luan Y., Song J., Fu G., Ge J., Zou Y., Zhang W. (2020). Insulin-Attenuated Inflammatory Response of Dendritic Cells in Diabetes by Regulating RAGE-PKC β 1-IRS1-NF- κ B Signal Pathway: A Study on the Anti-Inflammatory Mechanism of Insulin in Diabetes. J. Diabetes Res..

[58] Gao N., Yan C., Lee P., Sun H., Yu F.-S.S. (2016). Dendritic Cell Dysfunction and Diabetic Sensory Neuropathy in the Cornea. J. Clin. Investig..

[59] Rees D.A., Alcolado J.C. (2005). Animal Models of Diabetes Mellitus. Diabet. Med..

[60] King A.J.F. (2012). The Use of Animal Models in Diabetes Research. Br. J. Pharmacol..

[61] Mendez J.D., Ramos H.G. (1994). Animal Models in Diabetes Research. Arch. Med. Res..

[62] Chatzigeorgiou A., Halapas A., Kalafatakis K., Kamper E. (2009). The Use of Animal Models in the Study of Diabetes Mellitus. In Vivo.

[63] Kleinert M., Clemmensen C., Hofmann S.M., Moore M.C., Renner S., Woods S.C., Huypens P., Beckers J., De Angelis M.H., Schürmann A. (2018). Animal Models of Obesity and Diabetes Mellitus. Nat. Rev. Endocrinol..

[64] Center for Disease Control and Prevention (2022). National Diabetes Statistics Report.

[65] Wang S., Lai X., Deng Y., Song Y. (2019). Correlation between Mouse Age and Human Age in Anti-Tumor Research: Significance and Method Establishment. Life Sci..

[66] Harris M.I., Klein R., Welborn T.A., Knuiman M.W. (1992). Onset of NIDDM Occurs at Least 4–7 Yr before Clinical Diagnosis. Diabetes Care.

[67] Porta M., Curletto G., Cipullo D., De la Longrais R.R., Trento M., Passera P., Taulaigo A.V., Di Miceli S., Cenci A., Dalmasso P. (2014). Estimating the Delay between Onset and Diagnosis of Type 2 Diabetes from the Time Course of Retinopathy Prevalence. Diabetes Care.

[68] Liu G., Chen L., Cai Q., Wu H., Chen Z., Zhang X., Lu P. (2018). Streptozotocin-induced Diabetic Mice Exhibit Reduced Experimental Choroidal Neovascularization but Not Corneal Neovascularization. Mol. Med. Rep..

[69] Sun N., Yang G., Zhao H., Savelkoul H.F.J., An L. (2005). Multidose Streptozotocin Induction of Diabetes in BALB/c Mice Induces a Dominant Oxidative Macrophage and a Conversion of TH1 to T H2 Phenotypes during Disease Progression. Mediat. Inflamm..

[70] Neto A.F., Dell’Armelina Rocha P.R., Perez E.C., Xavier J.G., Peres G.B., Spadacci-Morena D.D., Alvares-Saraiva A.M., Lallo M.A. (2017). Diabetes Mellitus Increases the Susceptibility to Encephalitozoonosis in Mice. PLoS ONE.

[71] Wang H., Li H., Jiang X., Shi W., Shen Z., Li M. (2014). Hepcidin Is Directly Regulated by Insulin and Plays an Important Role in Iron Overload in Streptozotocin-Induced Diabetic Rats. Diabetes.

[72] Graham M.L., Janecek J.L., Kittredge J.A., Hering B.J., Schuurman H.-J. (2011). The Streptozotocin-Induced Diabetic Nude Mouse Model: Differences between Animals from Different Sources. Comp. Med..

[73] Tian L., Nikolic-Paterson D.J., Tesch G.H. (2019). Establishing Equivalent Diabetes in Male and Female Nos3-Deficient Mice Results in a Comparable Onset of Diabetic Kidney Injury. Physiol. Rep..

[74] Ryu Y., Kim Y.J., Kim Y.Y., Kim J., Kim S.W., Kim H., Ku S.Y. (2021). Consecutive Low Doses of Streptozotocin Induce Polycystic Ovary Syndrome Features in Mice. Int. J. Mol. Sci..

[75] Moore A., Shindikar A., Fomison-Nurse I., Riu F., Munasinghe P.E., Parshu Ram T., Saxena P., Coffey S., Bunton R.W., Galvin I.F. (2014). Rapid Onset of Cardiomyopathy in STZ-Induced Female Diabetic Mice Involves the Downregulation of pro-Survival Pim-1. Cardiovasc. Diabetol..

[76] Aaberg M.L., Burch D.M., Hud Z.R., Zacharias M.P. (2008). Gender Differences in the Onset of Diabetic Neuropathy. J. Diabetes Complicat..

