# Supplementary material for: Effects of Diabetes Mellitus on Corneal Immune Cell Activation and the Development of Keratopathy
Source: Cells. 2024 Mar 18;13(6):532. doi: 10.3390/cells13060532 (PMC10969384; doi:10.3390/cells13060532)
Supplement: Supplementary file 1 [file cells-13-00532-s001.zip › cells-2852346-supplementary.pdf]

Table S1. Flow cytometry antibodies used in the experiments

CD45	1:200	A20	APC-CY7	Biolegend
Ly-6C	1:200	HK1.4	PE	Biolegend
Ly-6G	1:200	18A	PerCPC5.5	Biolegend
CD11b	1:200	M1/70	BV785	Biolegend
Zombie UV	1:100		INDO-1 (Violet)	Biolegend
